# Enhanced whole exome sequencing by higher DNA insert lengths

**DOI:** 10.1186/s12864-016-2698-y

**Published:** 2016-05-25

**Authors:** Claudia Pommerenke, Robert Geffers, Boyke Bunk, Sabin Bhuju, Sonja Eberth, Hans G. Drexler, Hilmar Quentmeier

**Affiliations:** Leibniz Institute DSMZ-German Collection of Microorganisms and Cell Cultures, Inhoffenstrasse 7B, Braunschweig, 38124 Germany; Helmholtz Centre for Infection Research, Inhoffenstrasse 7, Braunschweig, 38124 Germany

**Keywords:** Whole exome sequencing, DNA insert size, Read coverage, Evenness score, Variant calling

## Abstract

**Background:**

Whole exome sequencing (WES) has been proven to serve as a valuable basis for various applications such as variant calling and copy number variation (CNV) analyses. For those analyses the read coverage should be optimally balanced throughout protein coding regions at sufficient read depth. Unfortunately, WES is known for its uneven coverage within coding regions due to GC-rich regions or off-target enrichment.

**Results:**

In order to examine the irregularities of WES within genes, we applied Agilent SureSelectXT exome capture on human samples and sequenced these via Illumina in 2 × 101 paired-end mode. As we suspected the sequenced insert length to be crucial in the uneven coverage of exome captured samples, we sheared 12 genomic DNA samples to two different DNA insert size lengths, namely 130 and 170 bp. Interestingly, although mean coverages of target regions were clearly higher in samples of 130 bp insert length, the level of evenness was more pronounced in 170 bp samples. Moreover, merging overlapping paired-end reads revealed a positive effect on evenness indicating overlapping reads as another reason for the unevenness.

In addition, mutation analysis on a subset of the samples was performed. In these isogenic subclones, the false negative rate in the 130 bp samples was almost double to that in the 170 bp samples. Visual inspection of the discarded mutation sites exposed low coverages at the sites flanked by high amplitudes of coverage depth.

**Conclusions:**

Producing longer insert reads could be a good strategy to achieve better uniform read coverage in coding regions and hereby enhancing the effective sequencing yield to provide an improved basis for further variant calling and CNV analyses.

## Background

During the past years whole exome sequencing (WES) has gained much popularity in research and diagnostics, as focussing on protein-coding regions reduces sequencing costs compared to whole genome sequencing (WGS) [1–4]. Concentrating on exonic regions minimises the sequencing target area of the human genome with about 3 Gbp to less than 2 % [3, 4]. There is a broad application area of WES such as variant calling [3–5] and analysis of copy number variations (CNV) [6, 7] demonstrating its general usefulness in the genomic field.

In terms of quality validation, many research efforts were focussing on the comparison of different exome enrichment platforms [8–11] and their performance to WGS techniques [12, 13]. Apparently, one recurrent bias appearing with WES is its inhomogeneous coverage across targeted protein-coding regions, which is suggested to be resolved by increasing read depth. However, increasing depth for WES would also place the economic costs for the alternative WGS in similar range to WES [12, 13]. For example, in one study the exome coverage of ≥ 20× needed an average of 160 × with WES, whereas WGS was sufficient with 44-56 × [12].

In the midst of this debate, we would like to propose a new aspect to the technical side of WES. During standard WES (2 × 101 paired-end sequencing) of our cancer cell lines, we observed a strong irregular distribution of read coverage along exons, which had a size of ≥1 kbp. In the following, the genomic DNA fraction sequenced as paired-end reads and flanked by illumina adapters is denominated as insert, in order to confine these to sequencing library fragments including illumina adapters. Calculation of the original DNA insert size revealed that the genomic DNA insert had a peak size of 130 bp. This prompted us to consider, whether longer purified DNA inserts might improve the evenness of read coverage. In the past, DNA insert lengths of 200–250 bp for 2 × 90 paired-end reads were mentioned to contribute to library optimisation, nonetheless, results were not shown [8]. Additionally, different fragmentation techniques improved coverage, yet DNA insert length were indicated for sonication (161 bp) but not for enzymatic fragmentation [10]. In another study short inserts of 100–200 bp are suggested instead of 500–800 bp [1], however, at that time the development of exome capture design just started and more importantly, very short single reads of 26 bp were analysed. Similarly, short reads of 35 and 50 bp were sequenced for a further publication, in which short insert sizes of 120 bp are recommended given the short median length of 120 bp of human exons [14]. Apparently, no specific study on the effect of DNA insert sizes to paired-end sequencing has been published to date.

Therefore, we tested in this study, whether the genomic DNA insert length influences the uniformity of read coverage within targeted regions. Beside samples with 130 bp peak insert length, a sample group with 170 bp peak inserts was produced and validated. The evenness score [14] was applied as metric for assessing the effectiveness of target region coverage. Altogether, we would like to provide a short technical note on the effect of DNA insert length on the evenness of coverage for paired-end sequenced reads.

## Results and discussion

### Production of WES libraries with two different insert sizes

**Fig. 1:**
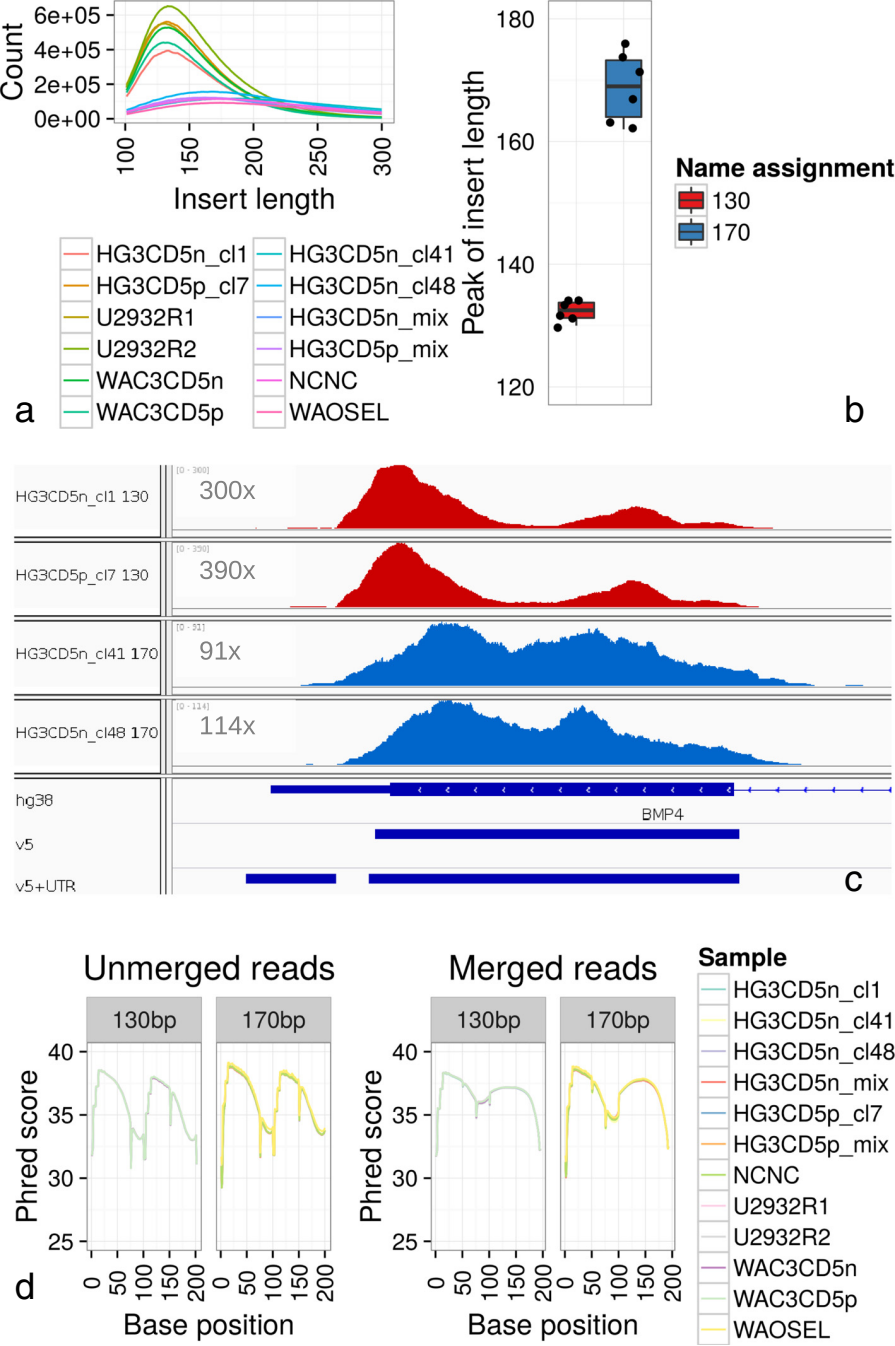
DNA shearing to 130 and 170 bp fractions before Illumina adapter ligation; sequencing base quality. **a** DNA insert length distribution per sample. **b** Peak insert lengths for the two different sample groups. **c** Alignment histograms for 130 bp insert samples (*red*) exhibited high amplitudes of coverage within the exon in comparison to 170 bp (*blue*) as exemplified by this gene BMP4 via IGV. Target regions of Agilent v5 and v5+UTR are given in the last two lines. Please note the 3 × fold higher maximum coverage of 130 bp samples. **d** High Phred score quality values for mapped paired-end reads. Base calling quality was high after trimming and mapping to the human genome. As expected, for both reads in forward and reverse direction (1–100 and 101–200 bases) read quality increased during the first 10 cycles and dropped gradually due to de-phasing errors of Illumina’s sequencing pipeline. After joining paired-end reads, quality scores improved between 75–125 cycles, as the best scores were kept while merging. Quality scores were ≥30 throughout nearly all cycles and similar between 130 and 170 bp samples

For exploring the effect of different genomic DNA insert lengths on the uniformity of coverage in whole exome sequencing (WES), we varied the DNA shearing by acoustic fragmentation (Covaris). Two DNA inserts groups of six samples each were yielded with approximately 130 and 170 bp length (Fig. 1a+b). Exome enrichment was conducted with Agilent SureSelectXT All Exon v5+UTR/v5 and sequencing produced 18–54 million reads per sample (see Table 1). After trimming, removing PCR duplicates and mapping the reads to the human GENCODE genome v21, manual inspection of alignments to many exons exceeding 1 kbp indicated large amplitudes of coverage for 130 bp compared to 170 bp samples (Fig. 1c). This observation, that longer inserts might compensate for “mountain-valley” profiles in coverage, encouraged us to examine this in detail.

**Table 1:** Portfolio of the samples in this study

Sample	Cell line*	Agilent	Insert	Mio.
		SureSelectXT	length bp	Reads
HG3CD5n_cl1	HG3	v5+UTR	134	32.4
HG3CD5p_cl7	HG3	v5+UTR	132	44.4
U2932R1	U-2932	v5+UTR	131	44.1
U2932R2	U-2932	v5+UTR	133	53.8
WAC3CD5n	WA-C3CD5+	v5+UTR	134	43.3
WAC3CD5p	WA-C3CD5+	v5+UTR	130	35.9
HG3CD5n_cl41	HG3	v5	176	25.9
HG3CD5n_cl48	HG3	v5	167	27.9
HG3CD5n_mix	HG3	v5	162	19.8
HG3CD5p_mix	HG3	v5	163	21.8
NCNC	NC-NC	v5	171	22.2
WAOSEL	WA-OSEL	v5	174	18.0

^*^All cell lines are held at the DSMZ

Since the mean DNA insert peak for each sample group with 130 and 170 bp, respectively, was smaller than the resulting paired-end sequenced reads of 2 × 101 bp, a high percentage of paired-end reads contained overlapping sequences. As these overlaps did not contain further information for e.g. variant calling and CNV analysis, paired-end reads were joined where overlapping sequences were found and aligned to the human genome. Trimming, read mapping and joining statistics are summarised in Table 2 and Phred quality scores for the sequencing cycles are visualised in Fig. 1d. Nearly all sequenced bases for further analyses had quality scores ≥30.

**Table 2:** Preprocessing statistics

	Trimming R1		Trimming R2		Mapped	Joined
Sample	reads	bases	reads	bases	reads	reads
HG3CD5n_cl1	19,9 %	9,0	22,4 %	22,2	93,7 %	80,5 %
HG3CD5p_cl7	20,0 %	8,8	22,4 %	22,1	93,6 %	82,4 %
U2932R1	20,1 %	8,9	22,1 %	21,8	93,5 %	81,9 %
U2932R2	20,0 %	8,9	22,6 %	22,8	93,7 %	80,1 %
WAC3CD5n	22,6 %	8,9	20,0 %	22,4	93,5 %	80,6 %
WAC3CD5p	20,0 %	9,0	22,4 %	21,8	93,7 %	81,6 %
HG3CD5n_cl41	14,6 %	12,8	12,7 %	43,1	90,5 %	38,0 %
HG3CD5n_cl48	14,5 %	12,6	12,5 %	42,7	90,3 %	47,1 %
HG3CD5n_mix	14,3 %	12,5	12,1 %	41,9	90,6 %	50,8 %
HG3CD5p_mix	13,7 %	12,3	11,9 %	42,4	91,0 %	46,8 %
NCNC	14,6 %	12,7	12,4 %	43,7	89,2 %	43,8 %
WAOSEL	13,3 %	15,9	10,3 %	42,4	88,0 %	44,4 %

In the following sections unmerged and merged sequences are compared along with contrasting 130 versus 170 bp insert results.

### Differences in exome capture and read depth

**Fig. 2:**
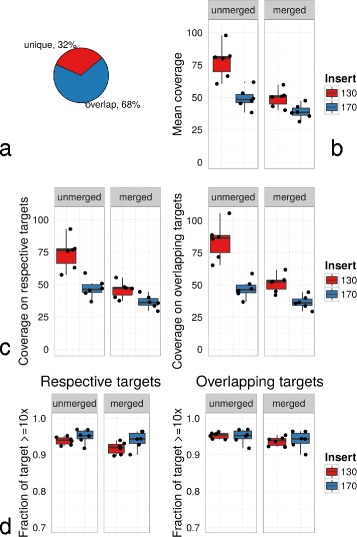
Target regions and relative read coverages. **a** Agilent SureSelectXT v5+UTR target regions (75 Mb) consisted of 68 % overlapping bases to v5 and a unique fraction of 32 %. The target region of v5 (50 Mb) was nearly fully contained in v5+UTR. **b** Mean coverage of unmerged and merged paired-end reads considering the size of respective target regions 75 and 50 Mbp for 130 bp and 170 bp, respectively. The average coverage was higher in 130 bp inserts than in 170 bp. This difference declined substantially when merging joint paired-end reads. **c** Recalculation of coverage of unmerged and merged paired-end reads on the respective specific target regions and on common target regions only. The mean coverage on the respective target regions was higher in 130 bp insert samples. **d** Portion of respective target regions covered by at least 10 ×. Despite higher coverage means for 130 bp, a smaller fraction of target regions was apparent at ≥10× depth for 130 bp samples. For overlapping target regions of v5 and v5+UTR the fraction of covered regions was still not higher in 130 bp reads as would be implicated by the higher coverage means

We applied two different exome enrichment kits in this study, namely Agilent SureSelectXT v5+UTR and v5 on the 130 bp genomic DNA insert and 170 bp group, respectively (see Table 1). The genomic target region both kits are covering were nearly identical except for the UTR stretches which were exclusively contained in v5+UTR: v5 target regions constitute 68 % of v5+UTR (75 Mb) and 99.9996 % of v5 (50 Mb) were included in v5+UTR (Fig. 2a). Hence, for further comparison the specific target region needed to be considered.

Although aiming to adapt the read numbers to the respective target regions during the sequencing process, the mean coverage to the target region sizes 75 and 50 Mbp was increased for 130 bp compared to 170 bp inserts (Fig. 2b). However, this difference attenuated when computing coverage means for merged paired-end sequences. Similar results were obtained when calculating the mean coverage of 130 and 170 bp inserts on their respective target regions and on overlapping target regions of v5 and v5+UTR (Fig. 2c). Intriguingly, concentrating on the fraction in the respective target region at ≥10× read depth revealed a smaller fraction of 130 bp samples covered at that mininum read depth than for 170 bp (Fig. 2d). Low coverages in turn mean impaired mutation detection in these regions, whereas at the same time excessive coverage of target regions in 130 bp samples (Fig. 1c) seems dispensible.

### Comparing uniformity of coverage

**Fig. 3:**
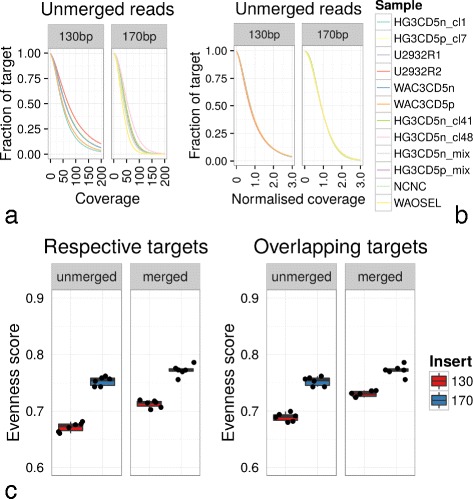
Evenness between different insert groups and unmerged/merged sequences. Before (**a**) and after (**b**) normalisation of coverage to the fraction of respective target regions for unmerged sequences. The complete integral of normalised coverage to the target region is summing up to 1. **c** The evenness score computed from the area under the curve of unmerged (Fig. 3 b) and merged sequences between 0 and 1 normalised coverage. The closer the evenness score is to 1, the better the uniformity of base coverage. The impact of higher insert length was evident; merged inserts gained top evenness scores regardless of relating to the specific corresponding target region or to overlapping target regions

As a measure for the skewed distribution of exome captured sequences in the coding regions, the evenness score was calculated allowing for target region and library size correction [14] (Fig. 3). Hereby, after normalising the coverage of each sample to the integral for each coverage curve to 1 (Fig. 3a+b), the area below the curve between 0 and 1 is defined as the evenness score [14]. Bearing in mind that the higher the evenness score, the more even the coverage, the evenness score for 170 bp insert samples was clearly higher than 130 bp and hence its coverage more even than the 130 bp group (Fig. 3c) despite the higher average coverage of 130 bp (Fig. 2c). Excluding overlapping sequences within the paired-end reads yielded a substantial rise in evenness for both insert groups.

Since using different enrichment kits for 130 and 170 bp samples respectively, the additional UTR target region fraction might be the cause of the unevenness observed in 130 bp inserts. However, the differences in the evenness scores were comparable to the results above when calculated on the common target region of v5 for both 130 and 170 bp inserts (Fig. 3c). The augmented evenness score for 130 bp inserts in the common coding target regions compared to its v5+UTR target region might even hint on inferior read coverage in the UTRs or inversely enhanced coverage (Fig. 2c) and uniformity for the coding regions.

For WES applying paired-end sequencing, libraries with small genomic DNA insert length produce overlapping sequences. These overlapping bases within one paired-end read provide no extra information on an alternate DNA strand or another allele, since they stem from the identical original genomic DNA sequence. The more overlapping bases within a paired-end read, the more bases remain unused, hence the effectivity to gain coverage shrinks with low insert sizes. Moreover, joining paired-end reads yielded in higher evenness scores particularly for 130 bp inserts (Fig. 3c), thereby showing another negative impact of redundant overlapping sequences.

On the other side, the median size of human exons is 120 bp, thus many bases will map off-target with longer inserts [14], however, the coverage in longer exons would reach higher uniformity and higher minimum depth (Fig. 2) instead of distinct “mountain-valley” coverage silhouette. Increasing coverage depth unfortunately would not yield in proportionally homogeneous coverage (Fig. 2 and [12]). Higher evenness in turn is prerequisite to effectively detect mutations, which is achieved with longer inserts (Fig. 3c). These longer inserts can be produced by a plain technical adjustment in the DNA sample fragmentation step. It may be speculated that peak library sizes of >200 bp will result in even better evenness and simultaneously minimise costly overlapping read bases and reduce excess coverages.

### Missing mutations in shorter insert samples

**Fig. 4:**
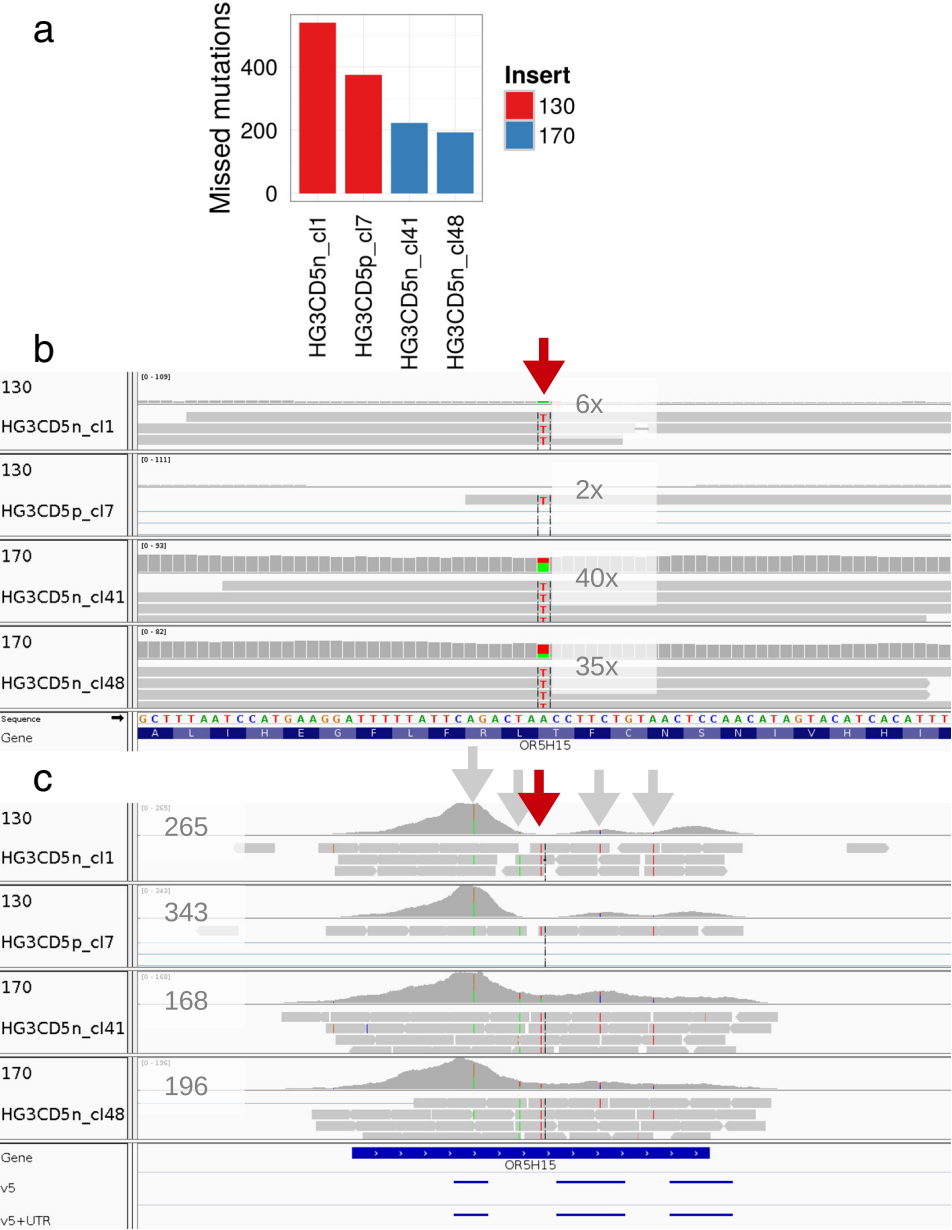
Missed mutations exemplified on four isogenic subclones. **a** The DNA of four isogenic subclones (human HG-3 cell line) were fragmented to 130 bp for HG3CD5n_cl1 and HG3CD5p_cl7 and to 170 bp peak insert sizes for HG3CD5n_cl41 and HG3CD5n_cl48. Several mutations were missed by variant calling for samples fragmented to 130 bp, but clearly less for 170 bp. **b** Example mutation on gene OR5H15 (*red arrow*) with coverage depth of 6 × and 2 × for 130 bp and 40 × and 35 × depth for 170 bp insert samples. OR5H15 does not contain any UTRs in this single 900 bp exon. Reads were sorted in IGV to bases at the mutation site, hence all detected Ts for 130 bp samples (3 and 1, respectively) are indicated. **c** Specific target regions of v5+UTR and v5 in gene OR5H15 were identical to which the coverage histogram peaks map. Target regions are given in the last two lines. The amplitudes were higher for the 130 bp samples as well as the maximum read depth in the visible region compared to 170 bp samples. At the same time pronounced amplitudes were also obvious for 170 bp within the gene region of OR5H15 implying that sequencing longer DNA fragments would have gained even smaller amplitudes and a higher coverage across the gene region. The four subclones carried four further mutations (*grey arrows*) beside the failed mutation of 130 bp samples (*red arrow*) indicating sequence similarity of the subclones

To demonstrate that shorter DNA inserts do increase the false negative rate for mutation analysis, we included four isogenic subclones of the human cell line HG-3. All four HG-3 subclones were sequenced at a comparable depth to their respective target regions. Of 9087 mutations found in at least one of the subclones, 223 and 193 were missed in the 170 bp samples but nearly twice as many mutations (540 and 375) in 130 bp (Fig. 4a), although coverage was slightly higher in 130 bp inserts for common target coding regions (Fig. 2c).

Manual inspection of 44 selected mutations with a minimum depth ≥20× in both 130 bp peak insert samples and simultaneously <10× in 170 bp samples and *vice versa* delivered mutations in four genes, that were discarded by longer insert samples, but mutations in 20 genes were missed by shorter inserts (e.g. Fig. 4b). Many of the failed mutations by the shorter inserts were found in regions, where amplitudes between maximum and minimum depth were high in the target regions forming a “mountain-valley” pattern as for the mutation in OR5H15 (Fig. 4c). Although this mutation dropped out in the shorter insert samples due to low depth in a “valley”, the biallelic nature seen in the longer insert was also true for shorter inserts. Here, higher coverage depth in target regions for 130 bp samples could not improve mutation detection in this gene. Expanding the insert length resulted in mapping more reads to off-target regions of OR5H15, which was more appropriate for this bait design.

The question arose whether the bait-balancing differences between the two exome enrichment kits may account for the skewed coverage of 130 bp compared to 170 bp samples. Bait-balancing is applied for adjusting the number of probes to the binding efficiency of the targeted regions. For OR5H15 as an example all coverage histogram peaks reflected the target regions, which were identical to v5 and v5+UTR (Fig. 4c). An unoptimised bait-balance between the given oligos within this single exon for both insert groups was apparent and the bait-balance of the v5 and v5+UTR kit was similar if not identical. It seems, that the manufacturer’s recommended 130–150 bp peak insert fragmentation was insufficient to span the entire annotated exon of OR5H15 on the basis of the target region rather than due to bait-balancing effects of the different capture kits used.

To overcome this technical shortcoming, the baits of the capture assay could be redesigned in closer proximity, or the potential of the current platform could be exploited by applying longer insert sizes. This would be a trade-off between too low as well as excessive read coverages and capturing off-target regions. Since flanking sequences to target regions often reside within annotated gene regions, achieving higher uniform coverage should be prioritised.

## Conclusions

Although widely used, one major drawback of WES is its skewed coverage distribution within the targeted exome. By simply enlarging genomic DNA fragment sizes before exome capturing, the evenness of coverage can be augmented. We think that WES with an average coverage of 80x in contrast to WGS will remain feasible for smaller studies with limited budget in the next years, therefore any optimisation of this technology is assumed to affect a broad community.

Hence, increasing the DNA insert length maybe even longer than 170 bp will gain better uniform read coverage for WES and thus provide an improved basis for variant calling and CNV analyses at minimised sequencing costs.

## Methods

### Samples, exome enrichment and sequencing

A selection of 2 × 6 human cancer cell lines (see Table 1) was prepared for WES all held by the DSMZ cell line bank (http://www.dsmz.de) and cultured as described previously [15].

Fragmentation of 100 ng purified genomic DNA (gDNA) in 55 *μ*l Tris-EDTA buffer was done on Covaris S2 and the procedure adjusted to obtain fragments with a peak length of 130 and 170 bp, respectively. After library preparation from 100 ng of fragmented gDNA using Agilent SureSelectXT v5 (50 Mb) and v5+UTR (75 Mb), libraries were purified, size validated and prepared for sequencing according to the manufacturer’s protocols. Libraries were sequenced on Illumina HiSeq2500 using TruSeq SBS Kit v3-HS (2 × 101, paired-end run). Concentration, quality, fragment sizes of purified genomic DNA (gDNA) and libraries were controlled by Agilent Technologies 2100 Bioanalyzer (Agilent Technologies; Waldbronn, Germany).

### Sequence processing, mapping and data analysis

Before mapping raw reads in fastq format, sequences were trimmed at the ends for low quality (<Q20) or adapter contamination by fastq-mcf of the ea-utils toolbox (version 1.1.2-686). Subsequently, trimmed reads were evaluated via FastQC (version 0.11.3, http://www.bioinformatics.babraham.ac.uk/projects/fastqc). For another branch of the pipeline trimmed reads were merged via fastq-join of ea-utils (version 1.1.2-686). Alignments of unmerged trimmed reads and merged reads were carried out with STAR (version 2.4.1d) [16] to the v21/hg38/GRCh38 assembly of the human reference genome. After conversion of sam files to sorted bam files by samtools (version 0.1.19), PCR duplicates were removed via Picard tools (version 1.121, http://picard.sourceforge.net).

For visual inspection of alignments the IGV was applied [17]. Coverage was calculated by bedtools2 (version 2.19.1) based on the target region design Agilent provided at https://earray.chem.agilent.com/suredesign for Agilent SureSelect All Exon v5 (S04380110) and Agilent SureSelect All Exon v5+UTR (S04380219). These files were converted to gencode v21 coordinates by the UCSC liftover tool and files (https://genome-store.ucsc.edu/). Insert sizes were determined by Picard tools. Graphs were created in the R/Bioconductor environment (http://www.bioconductor.org/) in particular applying ggplot2 [18]. For comparability the evenness score [14] served as metrics for the uniform coverage in the target regions.

Variant calling was conducted by VarScan 2 [19] to identify mutations in four subclones of the HG-3 cell line with a minimum depth of 10 × and 2 × for an alternative allele. The four subclones were HG3CD5n_cl1 and HG3CD5p-cl7 for 130 bp peak insert sizes and HG3CD5n_cl41 and HG3CD5n_cl48 for 170 bp (see Table 1). Afterwards, the Variant Effect Predictor (release 77, http://www.ensembl.org/info/docs/tools/vep/index.html) helped to concentrate on mutations in coding regions with severe consequences such as missense and stop gained effects.

Data were deposited at ArrayExpress under the accession number E-MTAB-4527.

## Abbreviations

CNV, Copy number variation; DSMZ, Deutsche Sammlung von Mikroorganismen und Zellkulturen, German Collection of Microorganisms and Cell Cultures; IGV, Integrative genomics viewer; UTR, Untranslated region; WES, Whole exome sequencing; WGS, Whole genome sequencing.
